# Choosing Psychiatry as a Career: Motivators and Deterrents at a Critical Decision-Making Juncture

**DOI:** 10.1177/070674371405900808

**Published:** 2014-08

**Authors:** Lesley Wiesenfeld, Susan Abbey, Sue Glover Takahashi, Caroline Abrahams

**Affiliations:** 1 Deputy Psychiatrist-in-Chief, Mount Sinai Hospital, Toronto, Ontario; Assistant Professor, Department of Psychiatry, University of Toronto, Toronto, Ontario; Associate Director, Post-graduate Medical Education, Department of Psychiatry, University of Toronto, Toronto, Ontario.; 2 Psychiatrist-in-Chief, University Hospital Network–Toronto General Hospital, Toronto, Ontario; Professor, University of Toronto, Toronto, Ontario.; 3 Director, Education and Research, Post-graduate Medical Education, University of Toronto, Toronto, Ontario; Associate Professor, Department of Public Health, University of Toronto, Toronto, Ontario.; 4 Director, Policy and Analysis, Post-graduate Medical Education, University of Toronto, Toronto, Ontario.

**Keywords:** psychiatry, medical education, residency, career choice, survey questionnaire, postgraduate training program, specialization, Canada

## Abstract

**Objective:**

To examine factors influencing the choice of psychiatry as a career between residency program application and ranking decision making.

**Methods:**

Using an online questionnaire, applicants to the largest Canadian psychiatry residency program were surveyed about the impact of various factors on their ultimate decision to enter psychiatry residency training.

**Results:**

Applicants reported that patient-related stigma was a motivator in considering psychiatry as a career, but that negative comments from colleagues, friends, and family about choosing psychiatry was a deterrent. Training program length, limited treatments, and insufficient clerkship exposure were noted as deterrents to choosing psychiatry, though future job prospects, the growing role of neuroscience, and diagnostic complexity positively influenced choosing psychiatry as a specialty. Research and elective time away opportunities were deemed relatively unimportant to ranking decisions, compared with more highly weighted factors, such as program flexibility, emphasis on psychotherapy, service– training balance, and training program location. Most applicants also reported continuing to fine tune ranking decisions between the application and ranking submission deadline.

**Conclusions:**

Stigma, exposure to psychiatry, diagnostic complexity, and an encouraging job market were highlighted as positive influences on the choice to enter psychiatry residency. Interview and information days represent opportunities for continued targeted recruitment activity for psychiatry residency programs.

## Abstract (fr)

**Objectif:**

Examiner les facteurs qui influencent le choix de la psychiatrie comme carrière entre la demande à un programme de résidence et l’évaluation de la prise de décision.

**Méthodes:**

À l’aide d’un questionnaire en ligne, les candidats au programme canadien le plus vaste de résidence en psychiatrie ont été interrogés au sujet de l’effet de divers facteurs ayant pesé sur leur décision finale de s’inscrire à la formation de résidence en psychiatrie.

**Résultats:**

Les candidats ont déclaré que les stigmates liés aux patients étaient un motivateur pour envisager la psychiatrie comme carrière, mais que les commentaires négatifs des collègues, des amis et de la famille sur leur choix de la psychiatrie étaient dissuasifs. La durée du programme de formation, les traitements limités, et l’exposition insuffisante à des stages cliniques ont été notés comme étant des désincitations à choisir la psychiatrie, mais les perspectives d’emploi futur, le rôle croissant des neurosciences, et la complexité diagnostique influençaient positivement le choix de la psychiatrie comme spécialité. Les possibilités de recherche et de stages dans d’autres universités ont été jugées relativement sans importance pour l’évaluation des décisions, comparativement à des facteurs qui pèsent plus lourd comme la flexibilité des programmes, l’accent mis sur la psychothérapie, l’équilibre service–formation, et l’emplacement du programme de formation. La plupart des candidats ont également déclaré continuer de préciser l’évaluation de leurs décisions entre la demande et l’échéance de la soumission de la décision.

**Conclusions:**

Les stigmates, l’exposition à la psychiatrie, la complexité diagnostique et un marché du travail encourageant ont été soulignés comme étant des influences positives sur le choix d’entreprendre une résidence en psychiatrie. Les journées d’entrevue et d’information représentent des possibilités d’activités continues de recrutement ciblé pour les programmes de résidence en psychiatrie.

Recruitment to psychiatry residency positions remains a challenge across Canada,^1^ the United States,^2^ and internationally,^3^ with positions left unfilled in national residency matching programs each year. Given the estimates of societal need,^4^ residency training programs, medical schools, and health service planners must better understand and respond to the determinants of medical students’ decisions to enter residency training in psychiatry. Current literature highlights several contributors to the recruitment challenge, including medical and pre-medical school influences, financial imperatives, patient and discipline stigma, and lifestyle implications.^5^ The psychiatry recruitment literature largely includes retrospective perspectives of current psychiatry resident trainees and junior undergraduate medical students earlier in their training.^6^–^8^

To enhance the understanding of career decision-making processes and contribute to a comprehensive recruitment strategy, we surveyed applicants to the University of Toronto Psychiatry Residency Program who participated in the 2012/2013 CaRMS Residency Program Orientation and Interview activities. Applicants were surveyed immediately following the residency program ranking submission deadline, to probe a cross-section of perspectives immediately proximal to the decision-making window for trainees. Given that applicants to psychiatry residency programs do not ultimately exclusively rank psychiatry programs, the goal was to better understand the influences on final ranking decisions.

## Methods

In March 2013, an online self-report survey was sent to all (*n* = 111) University of Toronto Psychiatry Residency Program applicants applying through CaRMS. Applicants were surveyed shortly after the ranking preference submission deadline for applicants and programs but prior to the match date or announcement of residency position assignments by CaRMS. This post-ranking submission and pre-match results day survey window was explicitly chosen to minimize undue concern about participation impacting program ranking decisions, elicit feedback proximal to the medical students’ reflective process regarding ranking choices, and eliminate potential bias related to the psychological impact of the actual match result. Participation was voluntary and anonymous. The University of Toronto PGME Office provided research design support and the University of Toronto Research Ethics Board provided research ethics approval.

Survey questions (Table 1) were informed by themes from existing literature and by feedback provided by applicants in pilot surveys from 2 previous cycles of post-match applicant surveys. Consequently the questions were designed to probe the following key potential impacts on selecting psychiatry for residency: psychiatry-specific exposure, information–interview day experience, residency program features, discipline and patient-related stigma, and unique aspects of psychiatric practice, compared with other disciplines.

**Clinical Implications**

- Stigma regarding the psychiatric profession and psychiatric illness should be addressed in undergraduate and post-graduate medical education to aid recruitment.
- Increased elective exposure and highlighting identified psychiatry motivators, such as employment opportunities, clinical complexity, and neuroscience focus may improve recruitment.
- Trainees continue to consider program and discipline choices between application and ranking submission, thus this remains a critical recruitment opportunity.

**Limitations**

- There may be additional influences on career choice that may not have been detected by this survey.
- Survey design focused on trainees considering psychiatry strongly enough to apply for psychiatry residency and does not include other graduating trainees.
- The response rate was 54%, and nonrespondent applicants surveyed may have differed in their commitment to psychiatry as a career.

## Results

Sixty (*n* = 60) respondents participated in the survey (54% response rate), with 97% (*n* = 58) of respondents completing all questions. Descriptive statistics and narratives were used to interpret the responses.

### Motivators and Deterrents

Respondents identified the emphasis of neuroscience, diagnostic complexity, patient stigma, and the favourable job market for psychiatry as motivating factors in choosing a career in psychiatry. Conversely, factors such as discipline stigma among colleagues and residency program length were rated as deterrents from choosing a psychiatry residency (Table 2).

Insufficient elective time exposure to both psychiatry itself and to psychiatry programs at specific universities was identified as a barrier to selecting psychiatry. Applicants indicated that earlier exposure to mental health careers, psychiatry residents, and psychiatry faculty during medical school were key factors in aiding career explorations and ranking decisions.

Most respondents indicated that the stigma of psychiatric illness and affected patients made them more interested in the discipline. However, applicants also identified varied amounts of support from their friends, family, and colleagues, ranging from encouraging to mixed responses. Through additional narrative responses available in the survey, applicants indicated that friends and family sometimes had a perplexed or disappointed response to trainees indicating an intention to rank psychiatry highly.

### Making the Choice: Timing and Pragmatics

Students were asked to retrospectively report on the degree to which they felt firmly decided on psychiatry as a career at the time of applying, which, in the Canadian matching process, is about 3 months before program and student selections and rank lists are due. Thirty-three per cent (*n* = 19) of applicants described themselves as having been firmly decided on both psychiatry and a particular program as their top choice when they applied 5 months pre-match. Twenty-eight per cent of applicants (*n* = 16) stated that they had decided on psychiatry as a career choice but were undecided about the specific training program they would ultimately rank or select prior to entering the tour of programs. And 36% (*n* = 21) reported at the time of applying that they had been strongly considering psychiatry while also strongly considering other disciplines and were thus still refining their career decisions during the CaRMS interviews and program visits.

When asked to rank-order various program features as most important to a final ranking decision, program flexibility, service–training balance, and geographic location were ranked most highly (Table 3). The least important decision-making features noted included the following: telepsychiatry and outreach exposure and residency salary and benefits. No respondents identified postgraduation job security, lifestyle, or income as barriers to strongly considering psychiatry.

## Discussion

Our study uniquely explores student perspectives regarding the choice of a career in psychiatry, proximal to the decision-making window. With this sampling approach, we were able to investigate applicant reflection while reducing recall decay about decisional influences, yielding insights that may guide undergraduate and post-graduate medical education approaches to both maximizing the overall psychiatry residency applicant pool and optimizing residency programs’ recruitment from interested, albeit undecided, applicants. While this survey approach excludes the perspectives of potential applicants who did not apply to our program, our match results and post-match follow-up suggest that various trainees often include our program in their application choices, even while also strongly considering a different psychiatry training program or another discipline. Other contributions to the literature address the more undifferentiated group of undergraduate trainees.

### The Role of Stigma

Stigma emerges as a complex and heterogeneous factor in trainees’ decision making. Applicant responses highlight that stigma can be viewed through the lens of marginalized patients and (or) a marginalized professional identity or discipline.^9^ This is consistent with informal trainee feedback and with literature regarding the psychiatric profession.^10^ For some psychiatry-leaning applicants, perceived stigma appears to generate motivation and career passion; for others, it may engender ambivalence or reticence toward actually ranking psychiatry as a career. The key message emerging from our findings is that a sophisticated approach to addressing mental health stigma in medical education is required to optimize interest in psychiatry and the decision to enter psychiatric training. During the CaRMS interview and information tours, students are comparing and contrasting careers, fit, and role models while also receiving feedback and guidance from friends and family. Targeted attention and interventions are needed to enable psychiatry-inclined trainees to pursue psychiatry without the barrier and burden of hidden factors limiting their choice of psychiatry as a career.

### Exposure to Psychiatry

Complementing existing knowledge and scholarship,^11^ our survey findings further emphasize the importance of early and broad exposure to psychiatry to capture interest and enable self-reflection regarding suitability for this discipline. Considering exposure and stigma together, exposure not only offers career fit reflection but also provides trainees with the experience to respond to the concerns of colleagues, friends, and family about career choice. Given the evidence highlighting the importance of mentoring and positive supervision experiences as influential in career choices,^12^ insufficient elective access, even to one institution, may conceivably influence not only program choice but also discipline choice.

### The Role of Residency Program Interview and Information Days

Responses regarding the timing of residency program ranking decision making indicate that applicants are genuinely considering their options about program and discipline choice during their interview tours of schools and programs. With only a minority of respondents certain of their likely rankings at the time of application, we hypothesize that there are considerable opportunities for post-graduate training programs to further enhance recruitment in their approach to the residency program application process, particularly given unmatched and unfilled spots. Thus, during the interview and program-showcasing processes, psychiatry program directors may wish to ensure that their messaging highlights program features that serve as key motivators, while directly addressing potential deterrents. Societal need, the job market, and the growing interface between psychiatry and neuroscience should be highlighted as positive features of our discipline. While the job market consideration may have geographic variation, barriers to entry remain relatively low for graduating psychiatrists in comparison with procedural specialties requiring substantial infrastructure.

## Conclusion

Our survey and synthesis advances our understanding of some important factors that impact recruitment to psychiatry by illustrating perspectives of applicants at the time of high-stakes career decision making. These findings have the potential to guide undergraduate and post-graduate mental health capacity building by identifying strategic foci to optimize recruitment activities at various points of opting in or out of considering psychiatry. While the deterrent of program length may not seem overtly modifiable by residency training programs, the evolving competency-based framework for residency education may offer some opportunities for change. Further scholarship is needed in complementary areas, most notably the optimization of medical school selection of psychiatry-inclined trainees to expand the pool of interested and skilled applicants to psychiatry. Finally, as is apparent from a broad mental health stigma literature, our findings underscore the importance of patient and discipline antistigma efforts as a critical component of capacity building for the mental health service needs of today and the future.

## Figures and Tables

**Table 1:** Examples of survey questions

How much have other colleagues, friends, and family members been in support of you considering psychiatry as a career?
As you approached the Canadian Resident Matching Service tour and sought more information about programs, which features were important to your final decision?
How much does the impact of stigma of psychiatric illness or patients affect your interest in training to become a psychiatrist?
With respect to recruitment and career exploration, what do you think would have been helpful to you with respect to exploring psychiatry as a career?
When you consider reasons that might deter you from a career in psychiatry, please rate (motivator, no impact, or deterrent) the impact of the following on your career decision making: stigma among colleagues, length of residency program, neuroscience interface, diagnoses seeming hard to define, limited treatments, emphasis on psychotherapy, job market, and concerns regarding personal safety?

**Table 2:** Motivators and deterrents that influenced applicants’ decision to pursue a career in psychiatry (*n* = 60)

Factor	Responses *n* (%)		
	Motivators	Neutral	Deterrents
Stigma amongst colleagues	13 (22)	23 (38)	20 (33)
Income	11 (18)	31 (52)	14 (23)
Program length (too long)	3 (5)	26 (43)	27 (45)
Emphasis on neuroscience	25 (42)	23 (38)	8 (13)
Diagnostic complexity	31 (52)	18 (30)	7 (12)
Limited treatments	20 (33)	25 (42)	11 (18)
Emphasis on psychotherapy	33 (55)	16 (27)	7 (12)
Job market for psychiatry	48 (80)	7 (12)	1 (2)
Concerns regarding personal safety	2 (3)	45 (75)	9 (15)

Applicants’ responses to the question: please rate the impact of these factors with respect to whether they deter you away from or motivate you toward choosing psychiatry as a career.

**Table 3:** Features of psychiatry residency programs ranked as the most important (ranked 1, 2, or 3 by applicants)

Feature	Responses, %
Program flexibility	78
Geographic location	53
Service-training balance	40
Formal mentorship	33
Research opportunities	25
Inner-city opportunities	18
Elective opportunities	15
Salary or benefits	10
Subspecialties	10
Support for electives	8
Outreach opportunities	3

## Abbreviations

CaRMS: Canadian Resident Matching Service
PGME: post-graduate medical education
